# Validation of T-MoCA in the Screening of Mild Cognitive Impairment in Chinese Patients With Atrial Fibrillation

**DOI:** 10.3389/fcvm.2022.896846

**Published:** 2022-06-22

**Authors:** Yiwei Lai, Manlin Zhao, Chao Jiang, Xin Du, Zhiyan Wang, Jingrui Zhang, Yu Bai, Baolei Xu, Weiwei Zhang, Ribo Tang, Caihua Sang, Deyong Long, Jianzeng Dong, Changsheng Ma

**Affiliations:** ^1^Department of Cardiology, Beijing Anzhen Hospital, Capital Medical University, National Clinical Research Centre for Cardiovascular Diseases, Beijing, China; ^2^Beijing Advanced Innovation Center for Big Data-Based Precision Medicine for Cardiovascular Diseases, Beijing, China; ^3^School of Clinical Medicine, Peking Union Medical College and Chinese Academy of Medical Science, Beijing, China; ^4^Department of Neurology, Beijing Anzhen Hospital, Capital Medical University, Beijing, China

**Keywords:** atrial fibrillation, mild cognitive impairment, Telephone interviewed Montreal Cognitive Assessment (T-MoCA), Mini-Mental Status Evaluation (MMSE), Clinical Dementia Rating (CDR)

## Abstract

**Background:**

Atrial fibrillation (AF) is associated with a high risk of mild cognitive impairment (MCI) and dementia. However, feasible and simple instruments that facilitate the regular assessment of cognitive status in patients with AF remain underdeveloped.

**Methods:**

Cognitive function was first evaluated using telephone Montreal cognitive assessment (T-MoCA), and then patients were invited for an in-person interview for cognitive assessment using both Clinical Dementia Rating (CDR) and mini-mental status evaluation (MMSE). Using CDR = 0.5 as a reference standard, the ability of T-MoCA and MMSE to discriminate cognitive dysfunction, stratified by education level, was tested by receiver–operating curve (ROC) analysis. The net reclassification index was calculated for comparison between the performance of T-MoCA and MMSE.

**Results:**

One hundred and one patients completed both telephone and in-person interview. Thirty-five MCI patients were identified as MCI using the criteria of CDR = 0.5. The areas under the ROC curve of T-MoCA were 0.80 (0.71–0.89), 0.83 (0.71–0.95), and 0.85 (0.64–0.92) for all patients, patients with high educational level, and patients with low education level, respectively. The optimal threshold was achieved at 16/17 with a sensitivity of 85.7% and a specificity of 69.7% in overall patients, 15/16 with a sensitivity of 88.2% and a specificity of 64.5% in the low educational level patients, and 16/17 with a sensitivity of 77.8% and a specificity of 87.9% in the high educational level patients. Compared to the criterion MMSE ≤ 27 and MMSE norms for the elderly Chinese community, the stratified T-MoCA threshold improves correct classification by 23.7% (*p* = 0.033) and 30.3% (*p* = 0.020), respectively.

**Conclusion:**

T-MoCA is a feasible and effective instrument for MCI screening in patients with AF.

## Introduction

Atrial fibrillation (AF) is one of the most common arrhythmias in adults, which remains to be a major public health challenge over the past decades (1). Cognitive dysfunction and dementia are one of the most concerning complications in patients with AF. The risk of dementia is 2-fold higher in patients with AF than that in the population without AF (2). Cognitive decline accelerates even in patients with AF at a young age when compared to those without AF (3). Mild cognitive impairment (MCI) is an early stage of cognitive decline, which is a crucial time for brain health protection. Therefore, regular cognitive status follow-ups for patients with AF are important for timely identification of those with mild cognitive impairment allowing subsequent preventions in clinical practice.

However, only a limited number of validated cognitive scales are available for MCI identification (4). Montreal Cognitive Assessment (MoCA) scale is the most widely used screening instrument for MCI. However, the MoCA scale requires an in-person interview, which limits the feasibility of regular follow-ups. Telephone interviewed Montreal Cognitive Assessment (T-MoCA) is a modified version of the MoCA scale. It has been validated as a reasonable screening tool for MCI in post-stroke patients in recent studies (5, 6). Although with reasonable sensitivity and specificity in screening MCI in patients with stroke, it has neither yet been validated externally in populations with different comorbidities nor has it been further validated in patients with different education levels. In the present study, we used the Chinese version of T-MoCA and examined its validity for MCI screening in Chinese patients with AF.

## Methods

### Study Design

The present study design conforms to the standards for reporting diagnostic accuracy studies (STARD) (7). Telephone cognitive assessment was first administered to patients with AF using T-MoCA 1–2 weeks before the in-person interview. Patients who have completed the telephone interview were invited for an in-person cognitive function assessment by another physician blinded to T-MoCA results using Clinical Dementia Rating (CDR) and Mini-Mental Status Evaluation (MMSE). Using CDR = 0.5 as a reference standard for MCI, the sensitivity and specificity of T-MoCA were analyzed in the overall population and educational subgroups. The performance of T-MoCA was also compared with the MMSE scale during MCI screening in patients with AF.

### Study Population

A telephone interview was administered in 145 consecutive patients in the waiting list for AF ablation in Beijing Anzhen Hospital, a tertiary medical center in Beijing, China from January 2019 to July 2019 by trained physicians using T-MoCA. The inclusion criteria were: (1) age ≥ 18 years; (2) atrial fibrillation diagnosed by 12-lead ECG or Holter monitoring; (3) willing to accept the cognitive assessment. Patients who met the following criteria will be excluded: (1) cannot cooperate well during telephone or in-person cognitive assessment; (2) any interventional or surgical procedure between telephone and in-person interview; (3) alcoholism or medications that may affect cognitive status; (4) history of neuropsychological diseases, such as Parkinson's disease, severe depression, any form of dementia, etc.

Among the preliminary enrolled patients, 9 did not finish the telephone interview (2 patients had hearing problems, 2 patients did not speak Mandarin, and 5 patients had other reasons). The other 136 patients completed the T-MoCA interview. Within this cohort, 28 patients received catheter ablation before the appointed time of in-person interview. To avoid the possible impact of catheter ablation on cognitive impairment (8), no further in-person interview was performed on those patients. Another 7 patients withdrew their consent to participate. In the end, 101 patients participated in an in-person cognitive interview within 2 weeks after the telephone interview.

### Data Collection

Baseline data on the history of hypertension, diabetes, coronary heart disease, heart failure, smoking, and alcohol use were collected in all patients during the in-person interview. Results of laboratory tests were extracted from a medical chart by a cardiologist.

### Telephone Interviewed Montreal Cognitive Assessment (T-MoCA)

The T-MoCA is a simplified version of MoCA from which trail making, visual structure, and naming are removed for the convenience of the telephone interview. It consists of digit span, attention, calculation, repetition, verbal fluency, abstraction, recall, and orientation, with a maximum score of 22. Since the Chinese version of MoCA has been widely validated and applied in previous reports (9, 10), the items of T-MoCA Chinese version are picked out from the MoCA, while the sequence and combination of the items in T-MoCA were kept the same as those in MoCA. Instructions for each item were strictly adhered to standardize the process of the interview.

### In-Person Cognitive Interview and Reference Standard

In-person cognitive interview was conducted by physicians blinded to T-MoCA results. Clinical Dementia Rating (CDR) was used for MCI diagnosis in the present study. CDR is a widely used semi-structured clinical measure for global cognitive status. It comprises six domains (memory, judgment and problem-solving, orientation, community affairs, home and hobbies, and personal care). The information in CDR is provided by assessing patients directly and by asking those who are familiar with the patient. All CDR raters were required to be certified (https://knightadrc.wustl.edu/CDR/CDR.htm). An online algorithm was used to calculate CDR scores (https://biostat.wustl.edu/~adrc/cdrpgm/index.html). CDR = 0.5 is a widely accepted MCI diagnostic criteria in Chinese-speaking populations (11, 12) and is also applied in previous T-MoCA validations (5, 6). It comprehensively reflects the patient's complaint of cognitive decline, objective cognitive deficit, and functional status, which conforms well to the revised Petersen's criteria for MCI diagnosis (13). Therefore, we used CDR = 0.5 as the reference standard for T-MoCA validation.

The Mini-Mental Status Evaluation (MMSE) is a widely used scale for screening cognitive impairment (14). Although limited by low sensitivity, it is commonly used by clinicians as it is easy to apply (4). The threshold of MMSE for MCI varies in different studies (15). A recently published data for the Chinese community elderly population proposed a stratified criterion (27/28 for those with a ≥7 years of education, 24/25 for those with a 1–6 years of education, and 19/20 for illiterates) (16). In the present study, we used this criterion as a comparison to the T-MoCA in MCI screening.

### Statistical Analysis

Data analysis was conducted using R3.5.1. Normative data were presented as mean [SD] and compared using the *t*-test, while non-normative data were presented as median [interquartile range] and compared using the Mann–Whitney U test. Categorical data were presented as count (frequency) and compared using the Chi-square test or Fisher's exact test.

The pROC package was used for the receiver operating curve (ROC) analysis and De Long's test was used to compare the area under the curve (AUC). Sensitivities and specificities for different thresholds were calculated in the overall patients and in patients with low and high educational levels respectively. The optimal threshold was defined as the score with the highest Youden index among all scores with sensitivity>75% and specificity >60%. When reasonable sensitivity and specificity cannot satisfy simultaneously, the optimal threshold was simply determined by the Youden index. The net reclassification index (NRI) of optimal thresholds of T-MoCA against the optimal threshold of MMSE in the present study and optimal thresholds suggested by the MMSE normative data of the Chinese community elderly population were calculated to assess whether and to what extent T-MoCA outperforms MMSE in screening MCI.

The sample size is estimated by PASS 15.0 prior to the study. Under the null hypothesis of a sensitivity of 0.5, a minimum of 13 cases has 0.8 the power to detect a sensitivity of 0.82 in the previous study at a significance level of 0.05. A minimum of 36 cases account for 0.9 power to detect a sensitivity of 0.75 under the null hypothesis of a sensitivity of 0.5 and a significance level of 0.05. Therefore, assuming the prevalence of MCI in the AF population is 30-40%, a sample size of 100 patients was considered.

Considering T-MoCA has only been validated in patients with stroke, which is an important but not the only mechanism of cognitive impairment in patients with AF, sensitivity analysis was conducted in patients without a history of stroke.

## Results

### Characteristics of Patients With MCI and With Normal Cognitive Function

**Figure 1:**
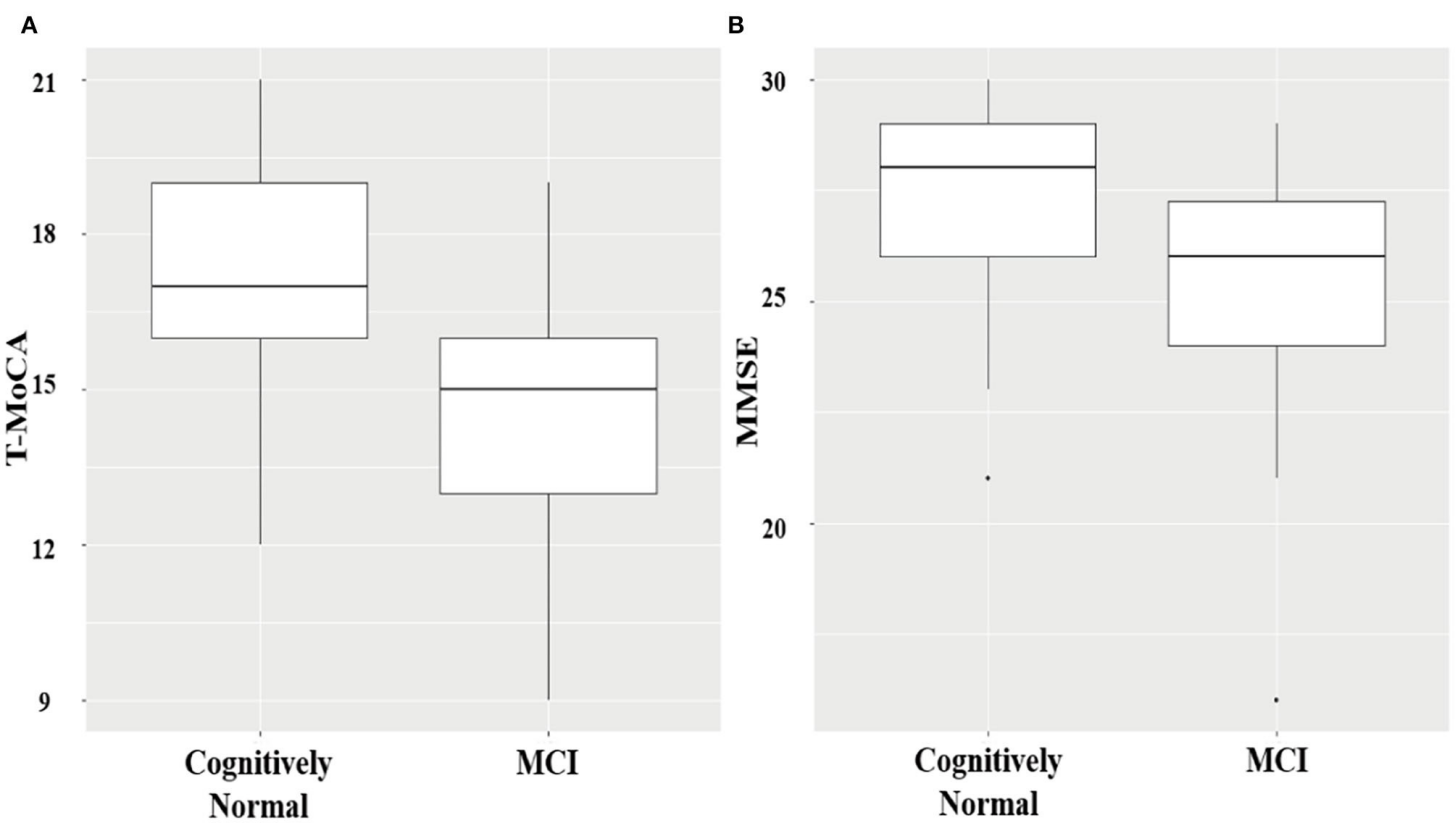
Boxplots showing the distribution of T-MoCA **(A)** and MMSE scores **(B)** in MCI and cognitively normal patients.

Among the 101 patients with AF receiving both T-MoCA interviews and in-person assessments, 35 patients were identified as MCI by in-person CDR assessment. Patients with and without MCI were not significantly different in age, sex, education level, medical history, smoking and drinking habit, left atrial diameter, left ventricular ejection fraction (LVEF), and CHA_2_DS_2_-VASc score (Table 1). T-MoCA scores in patients with and without MCI were 15 [13–16] and 17 [16–19], respectively (*p* < 0.001). MMSE scores in patients with and without MCI were 26 [24–27.5] and 28 [27–29], respectively (*p* < 0.001) (Figure 1). After adjusting for age, sex, education, type of AF, and CHA_2_DS_2_-VASc score both T-MoCA and MMSE score were negatively associated with MCI (OR = 0.48 (0.35–0.66) for T-MoCA and OR = 0.63 (0.49–0.80) for MMSE).

**Table 1:** Demographics of MCI and cognitively normal participants.

	**CDR = 0.5** **(*n* = 35)**	**CDR = 0** **(*n* = 66)**	**P-value**
Male gender	20 (57.1)	40 (60.6)	0.736
Age, years	62.8 [7.5]	61.3 [8.7]	0.388
Senior high school or above	18 (51.4)	35 (53.0)	0.878
Paroxysmal AF	25 (71.4)	49 (59.1)	0.221
Hypertension	25 (71.4)	38 (57.6)	0.171
Heart failure	4 (11.4)	6 (9.1)	0.735
Diabetes	8 (22.9)	17 (25.8)	0.748
CHD	8 (22.9)	11 (16.7)	0.449
Stroke	6(17.1)	4 (6.1)	0.091
Smoking	14 (40.0)	27 (40.9)	0.929
Drinking	7 (20.0)	14 (21.2)	0.886
Left atrial diameter, mm	38.4 [3.9]	39.0 [5.6]	0.529
Ejection fraction, %	60.9 [6.7]	61.9 [4.9]	0.398
CHA_2_DS_2_-VASc	2 [1–3]	2 [1–4]	0.148

*Data are mean [SD], median [interquartile range], or n (%). AF, atrial fibrillation; CHD, coronary heart disease*.

### Sensitivity and Specificity of T-MoCA and MMSE

The area under the curve for T-MoCA was 0.80 (0.71–0.89). Sensitivity and specificity for different thresholds are shown in the supplement (Supplementary Table 1). The optimal threshold of 16/17 in the overall population had a sensitivity of 85.7% and specificity of 69.7%. Stratifying the patients by educational level increased the AUC to 0.83 (0.71–0.95) in patients with low educational levels and 0.85 (0.64–0.92) in patients with high educational levels, respectively. The optimal threshold 16/17 had a sensitivity of 77.8% and specificity of 88.6% in the high educational level group, and the optimal threshold 15/16 had a sensitivity of 88.2% and specificity of 64.5% in the low educational level group (Table 2).

**Table 2:** Optimal thresholds of T-MoCA and corresponding parameters in the overall population and educational level subgroups using CDR as the reference standard.

	**Optimal** **threshold**	**Sensitivity**	**Specificity**	**Positive** **predictive** **value**	**Negative** **predictive** **value**
Overall (*n* = 101)	16/17	85.7	69.7	60.0	90.2
Low educational level (*n* = 48)	15/16	88.2	64.5	60.0	91.3
High educational level (*n* = 53)	16/17	77.8	88.6	77.8	88.6

**Figure 2:**
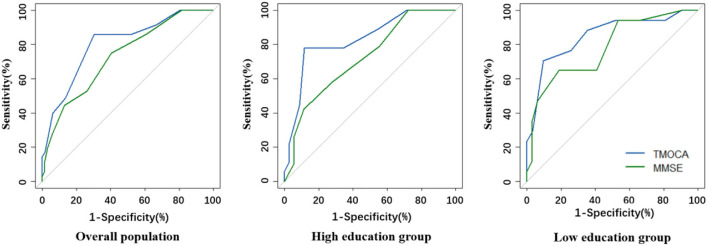
Comparison of ROC curves for T-MoCA and MMSE in the overall population and different educational groups.

The AUCs of MMSE were 0.75 (0.63–0.83), 0.72 (0.58–0.85), and 0.80 (0.64–0.92) in the overall study population, in patients with a high educational level, and in patients with a low educational level respectively, not significantly different with that of T-MoCA (Figure 2). The optimal sensitivity (74.2%) and specificity (62.1%) of MMSE for screening MCI were achieved at a threshold of 27/28 in the overall population. A sensitivity of 64.7% and a specificity of 86.7% were achieved in patients with a low educational level at an optimal threshold of 25/26. However, the sensitivity is too low for MMSE to be a screening tool in the high educational level group (Supplementary Table 2).

### Net Reclassification Improvement (NRI)

Compared with the optimal threshold of MMSE (≤ 27) in the present study, the stratified T-MoCA threshold (<16 for the low educational level group and <17 for the high educational level group) improved correct classification by 23.7% (*p* = 0.033). Stratified (NRI = 0.303, *p* = 0.020) thresholds also significantly improved classification when comparing with MCI criteria from MMSE norms of Chinese community elderly patients (16) (Table 3) Noteworthily, in 8 patients with MMSE ≥ 27 but correctly classified as MCI by T-MoCA, 7 of them had completed high school or above.

**Table 3:** Net reclassification index for T-MoCA by MMSE criteria for MCI.

	**Stratified T-MoCA**		
	**(16/17for high education**		
	**and 13/14 for low education)**		
	**NRI**	**Z**	***P*-value**
MMSE ≤ 27	0.237	1.84	0.033
Norms of Chinese community elder population*	0.303	2.05	0.020

*An education stratified MCI criteria in MMSE norms of Chinese community elderly population, which is 27/28 for ≥7 years of education, 24/25 for 1–6 years of education, and 19/20 for illiterates. T-MoCA, telephone Montreal cognitive assessment; MMSE, mini-mental state.

### Sensitivity Analysis

We further excluded 11 patients with previous stroke or TIA. In this non-stroke AF population, optimal T-MoCA thresholds for MCI remained the same, with an AUC of 0.80 (0.70–0.89) in the overall population and 0.82 (0.67–0.97), 0.84 (0.72–0.96) in the low and high educational groups, respectively (see Supplementary Table 3, supplement content, which shows the result of sensitivity analysis).

## Discussion

Among these consecutively enrolled patients on the AF ablation waiting list, the prevalence of MCI is 35%. This high prevalence underscores the necessity of timely identification of MCI to prevent or reverse the cognitive deterioration. Our study expanded the validation of T-MoCA, a simple telephone cognition assessment tool, to be used in MCI screening in patients with AF. T-MoCA improved the correct classification by at least 23% compared to MMSE.

MoCA score highly depends on cultural background. The optimal threshold for MCI is 26 in studies using the English version (17), whereas the optimal threshold is much lower in studies using the Chinese and Korean versions (9, 18). Similarly, in the present study, the optimal threshold of T-MoCA in the overall population was 2 points lower than that in previous studies (5, 6). We also noticed that the optimal threshold in the low education group is 1 point lower than that in the high education group. This highly conforms to the clinical MoCA assessment in which 1 additional score is added to patients with <12 years of education, and unlike the ceiling effect in MMSE, T-MoCA has reasonable sensitivity and specificity in both the low and high education groups. In the sensitivity analysis, parameters of ROC curves for non-stroke patients with AF remained almost unchanged, demonstrating that T-MoCA can also be applied in patients without a history of stroke.

The rationale for the clinical use of T-MoCA is embodied in several aspects. Firstly, it only takes about 10 min to complete the test, rendering it widely accepted by both patients and physicians. Secondly, T-MoCA can be easily accessed remotely by those unable to attend an in-person interview. These patients are often elderly, frail, or actively disabled, which are all independent factors associated with cognitive impairment (19, 20), and should therefore be followed-up more closely. Excluding these patients from the clinical study would favor selection bias and underestimate the prevalence of MCI in the population. Meanwhile, the telephone interview is highly cost-effective and can greatly reduce the loss to follow-up in clinical studies, particularly large ones with long-term follow-up. Thirdly, although there are many other telephone cognitive assessment tools, the majority were developed based on Alzheimer's MCI/dementia population and have a greater emphasis on memory (21). However, the mechanism of AF-associated cognitive impairment is more complex. MCI in patients with AF is more likely to be of cerebrovascular origin (22, 23), with multiple brain lesions, including cerebral small vessel disease and ischemic cerebral lesions (24). These lesions typically impair attention and executive function (25). T-MoCA is derived from a post-stroke population that may have a similar pathophysiological background to AF. Moreover, T-MoCA comprises various items that assess various cognitive domains. Delayed recall reflects episodic memory that is typical of Alzheimer's MCI/dementia (26), while items that reflect attention, working memory, processing speed, and language are necessary for vascular MCI/dementia assessment (27). However, the extrapolation of T-MoCA in a general population still remains further study in the future.

Despite the stress on comprehensive management for patients with AF in guidelines (28), primary and secondary prevention of MCI is often neglected. Patients with AF have a higher risk of MCI and dementia, which in turn affects the prognosis of AF, generating a vicious circle. Effective anticoagulation has been reported to be associated with alleviation of cognitive impairment in prospective cohorts (29–31). Catheter ablation, albeit possibly associated with temporary cognitive impairment after the procedure (8), is associated with lower risk of dementia in the long term (32). Physical exercise is reported to be associated with improved cognition trajectories and reduced brain lesions in patients with MCI (33). In recent years, several cognitive training systems have been developed and are recommended for clinical use to maintain cognitive status or even reverse MCI (34).

There are several limitations to the present study. Forty-four patients who answered the telephone interview were excluded from the study. This may cause a bias in prevalence estimation. Second, we did not administer detailed multiple neuropsychological testing for different cognitive domains to patients because some patients with severely symptomatic AF cannot tolerate a more than 1-h cognitive overload, while CDR comprises partly of informers' inquiry and may be more tolerable for its shorter patient testing. Besides, the practice effect may exist when T-MoCA is applied repeatedly, and so we are not able to examine the test–retest and inter-rater reliability of the scale. The present study only enrolled in-hospital patients and all of them received guideline-recommended therapy for AF and other cardiovascular risk factors, so we were not able to assess the effect of cognitive screening on prompt optimal patients' care.

## Conclusion

Our study showed that T-MoCA is an effective cognitive test that performs better than MMSE for MCI screening in patients with AF who are at high risk of cognitive impairment. The T-MoCA is simple to use and is able to be administered remotely. It may facilitate clinical cognitive screen in patients with AF, especially for general practitioners and cardiologists, and may further arouse more attention to cognitive status in the clinical management of AF.

## Data Availability Statement

The raw data supporting the conclusions of this article will be made available by the authors, without undue reservation.

## Ethics Statement

The studies involving human participants were reviewed and approved by Beijing Anzhen Hospital. The patients/participants provided their written informed consent to participate in this study.

## Author Contributions

YL, CJ, CS, and CM was responsible for the conception and design of the study. YL translated T-MoCA into simplified Chinese. MZ and JZ conducted the telephone interview. YL and ZW conducted the in-person interview. BX and WZ supervised the process of cognitive assessment and MCI diagnosis. RT, DL, and JD was responsible for AF diagnosis and interpretation of clinical data. YL, CJ, YB, and MZ drafted the manuscript. XD, CS, and CM critically appraised the manuscript and approved the final version. All authors have the access to all of the data, read the manuscript and agreed to be accountable for all aspects of the work.

## Funding

This work was supported by the National Key Research and Development Program of China (2020YFC2004803) and the National Natural Science Foundation of China (Grant Nos. 82100326 and 81900449), and grants from the Beijing Municipal Science and Technology Commission (D171100006817001 and Z181100001618011) and the Beijing Municipal Education Commission (KM202210025012).

## Conflict of Interest

CM has received honoraria from Bristol-Myers Squibb (BMS), Pfizer, Johnson & Johnson, Boehringer-Ingelheim (BI), and Bayer for giving lectures. JD has received honoraria from Johnson & Johnson for giving lectures. The remaining authors declare that the research was conducted in the absence of any commercial or financial relationships that could be construed as a potential conflict of interest.

## Publisher's Note

All claims expressed in this article are solely those of the authors and do not necessarily represent those of their affiliated organizations, or those of the publisher, the editors and the reviewers. Any product that may be evaluated in this article, or claim that may be made by its manufacturer, is not guaranteed or endorsed by the publisher.

## Abbreviations

AF: Atrial fibrillation
CDR: Clinical dementia rating
MCI: Mild cognitive impairment
MMSE: Mini-mental state evaluation
MoCA: Montreal cognitive assessment
NRI: Net reclassification index
ROC: Receiver operating curve
T-MoCA: Telephone Montreal cognitive assessment.
