# Supplementary material for: Validation of T-MoCA in the Screening of Mild Cognitive Impairment in Chinese Patients With Atrial Fibrillation
Source: Front Cardiovasc Med. 2022 Jun 22;9:896846. doi: 10.3389/fcvm.2022.896846 (PMC9257241; doi:10.3389/fcvm.2022.896846)
Supplement: Supplementary file 1 [file Data_Sheet_1.pdf]

Supplementary Material

Supplementary Table 1. Sensitivities and specificities of different T-MoCA thresholds for MCI

**Thresholds For T-MoCA**	**Overall population (n=101)**		**Low educational level group(n=48)**		**High educational level group(n=53)**	
	**Sensitivity**	**Specificity**	**Sensitivity**	**Specificity**	**Sensitivity**	**Specificity**
**18/19**	91.4	33.3	94.1	19.4	88.9	45.7
**17/18**	85.7	48.5	94.1	29.0	77.8	65.7
**16/17**	**85.7**	**69.7**	94.1	48.4	**77.8**	**88.6**
**15/16**	65.7	78.8	**88.2**	**64.5**	44.4	91.4
**14/15**	48.6	86.4	76.5	74.2	22.2	97.1
**13/14**	40.0	93.9	70.6	90.3	11.1	97.1

Supplementary Table 2. Sensitivities and specificities of different MMSE thresholds for MCI

Thresholds For MMSE	Overall population (n=101)		Low educational level group(n=48)		High educational level group(n=53)	
	Sensitivity	Specificity	Sensitivity	Specificity	Sensitivity	Specificity
**28/29**	85.7	40.9	94.1	36.7	77.8	45.7
**27/28**	**74.2**	**62.1**	94.1	50.0	55.6	74.3
**26/27**	54.3	75.8	64.7	63.3	44.4	88.6
**25/26**	45.7	89.4	**64.7**	**86.7**	27.8	94.3
**24/25**	28.6	95.5	47.1	96.7	11.1	94.3

Supplementary Table 3. Parameters of ROC curves for T-MoCA in sensitivity analysis

	AUC(95%CI)	Optimal threshold	Sensitivity	Specificity
**Overall**	0.80(0.70-0.89)	16/17	86.2	69.4
**Low education**	0.82(0.67-0.97)	15/16	84.6	65.5
**High education**	0.84(0.72-0.96)	16/17	81.3	87.9
